# A Rare Case of Intravascular Lobular Capillary Hemangioma Mimicking a Testicular Tumor: Diagnostic Challenges

**DOI:** 10.7759/cureus.70853

**Published:** 2024-10-04

**Authors:** Ahmet Erbagci, Abdullah Aydin, Ayşe Nur Toksöz Yıldırım, Bengu Cobanoglu Simsek

**Affiliations:** 1 Pathology, Istanbul Medeniyet University, Istanbul, TUR; 2 Pathology, Göztepe Research and Training Hospital, Istanbul, TUR

**Keywords:** case report, benign vascular lesion, testicular tumor mimicry, diagnostic challenges, intravascular lobular capillary hemangioma

## Abstract

Intravascular lobular capillary hemangioma (ILCH), also known as intravenous pyogenic granuloma, is a benign vascular lesion with a distinctive lobular arrangement of capillaries. It is typically confined to the head, neck, and upper extremities, and its occurrence in the testicular region is exceedingly rare. Here, we present a case of a 68-year-old male who was initially diagnosed with a testicular tumor based on imaging studies but was later found to have an ILCH located in the epididymis. This case underscores the diagnostic challenges posed by this rare entity due to its unusual presentation and mimicking of malignant conditions.

## Introduction

Intravascular lobular capillary hemangioma (ILCH) is a rare benign vascular tumor characterized by a proliferation of capillaries within the lumen of a vein. ILCH was first described by Cooper et al. in 1979 [1]. The pathogenesis of ILCH remains unclear, although trauma, hormonal changes, and infections have been implicated as contributing factors [2]. Most cases are reported in the head, neck, or upper extremities, and its presentation in the testicular region is exceedingly rare [3-5]. ILCH can affect a wide range of vascular beds, making its clinical presentation highly variable [6]. It is often misdiagnosed preoperatively due to its non-specific clinical and imaging features, which can mimic malignant vascular tumors [7]. We report a case of ILCH in the epididymis of a 68-year-old male, which was initially suspected to be a testicular malignancy.

## Case presentation

**Figure 1:**
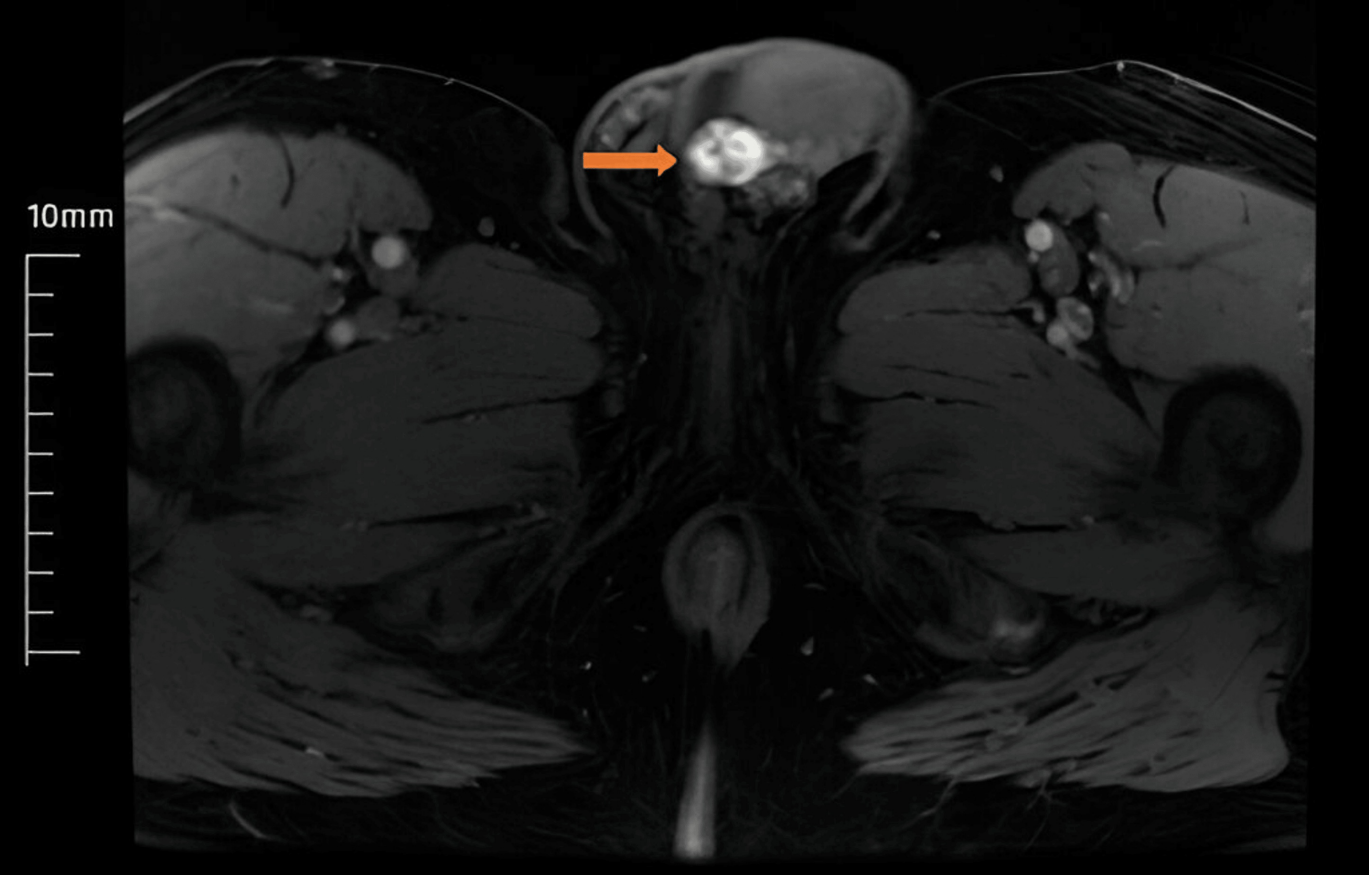
Contrast-enhanced MRI showing a hypervascular lesion (indicated by arrow).

A 68-year-old male presented to our clinic with a painless, palpable mass in the left testis. He had no history of trauma, infection, or significant testicular pathology. Scrotal Doppler ultrasound and MRI revealed a hypervascular lesion extending from the head of the epididymis to the testis, raising concerns for a malignant testicular tumor (Figure 1). Serum tumor markers, including alpha-fetoprotein (AFP), human chorionic gonadotropin (HCG), and lactate dehydrogenase (LDH), were within normal limits. Based on imaging findings, a preliminary diagnosis of testicular malignancy was made, and the patient underwent a left radical orchiectomy.

**Figure 2:**
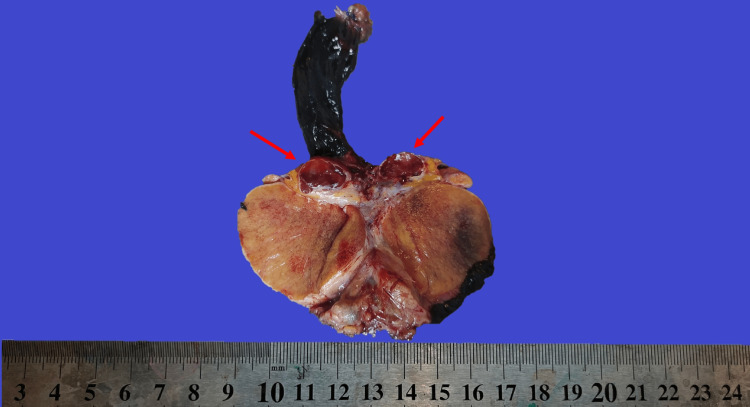
Macroscopic view A 1.7-cm red, homogeneous, intravascular mass is observed (indicated by arrow).

Gross examination of the specimen revealed no abnormalities in the testis itself. However, a 1.7 x 1.5 x 1 cm homogenous, red mass was found in the head of the epididymis, located intravascularly (Figure 2). The lesion's appearance was unlike typical testicular tumors, prompting further histopathological examination.

**Figure 3:**
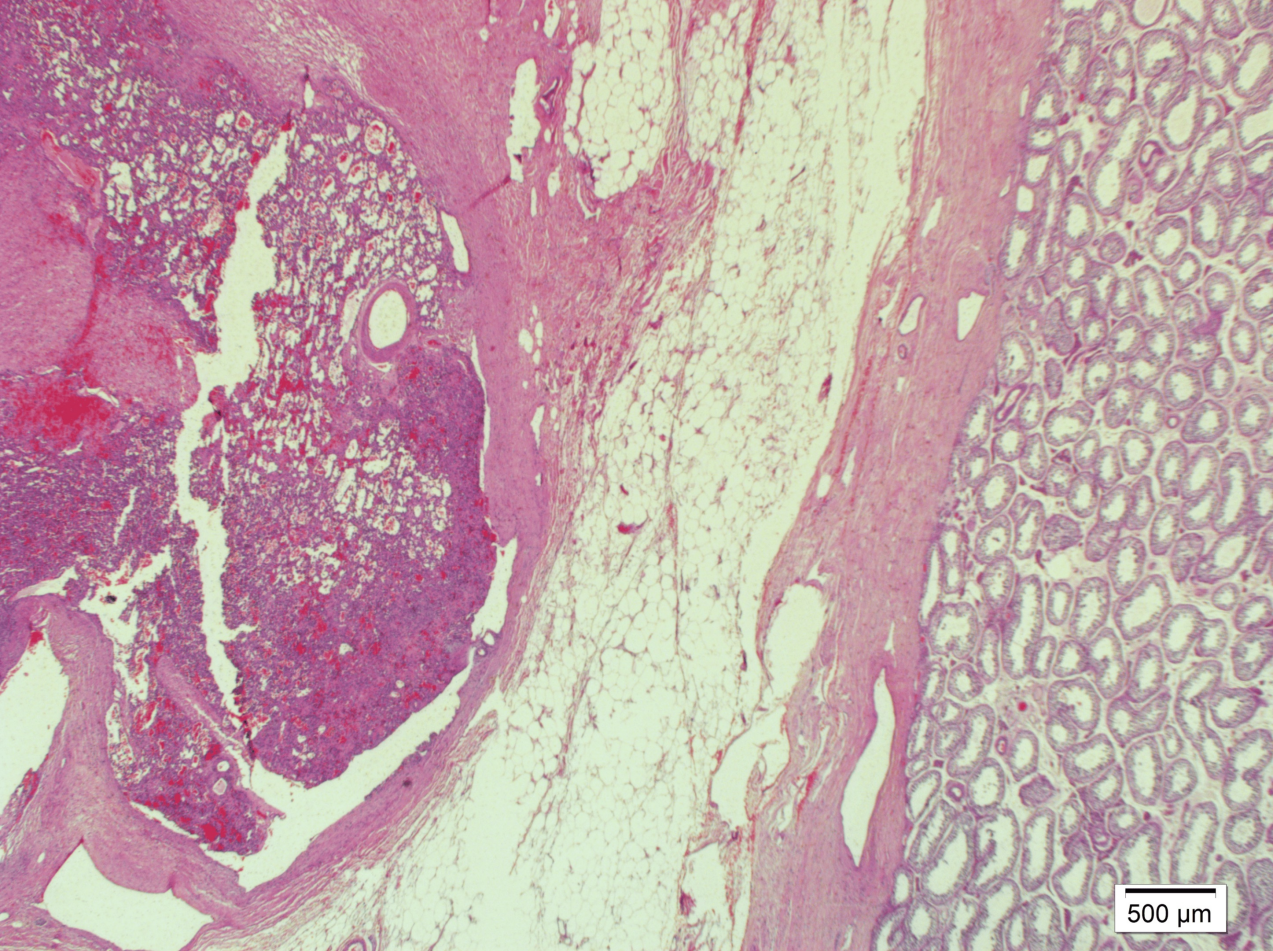
ILCH and testis Hematoxylin and eosin staining at x20 magnification shows normal testis on the right side, while ILCH composed of dilated vascular structures is observed on the left side. ILCH, intravascular lobular capillary hemangioma

**Figure 4:**
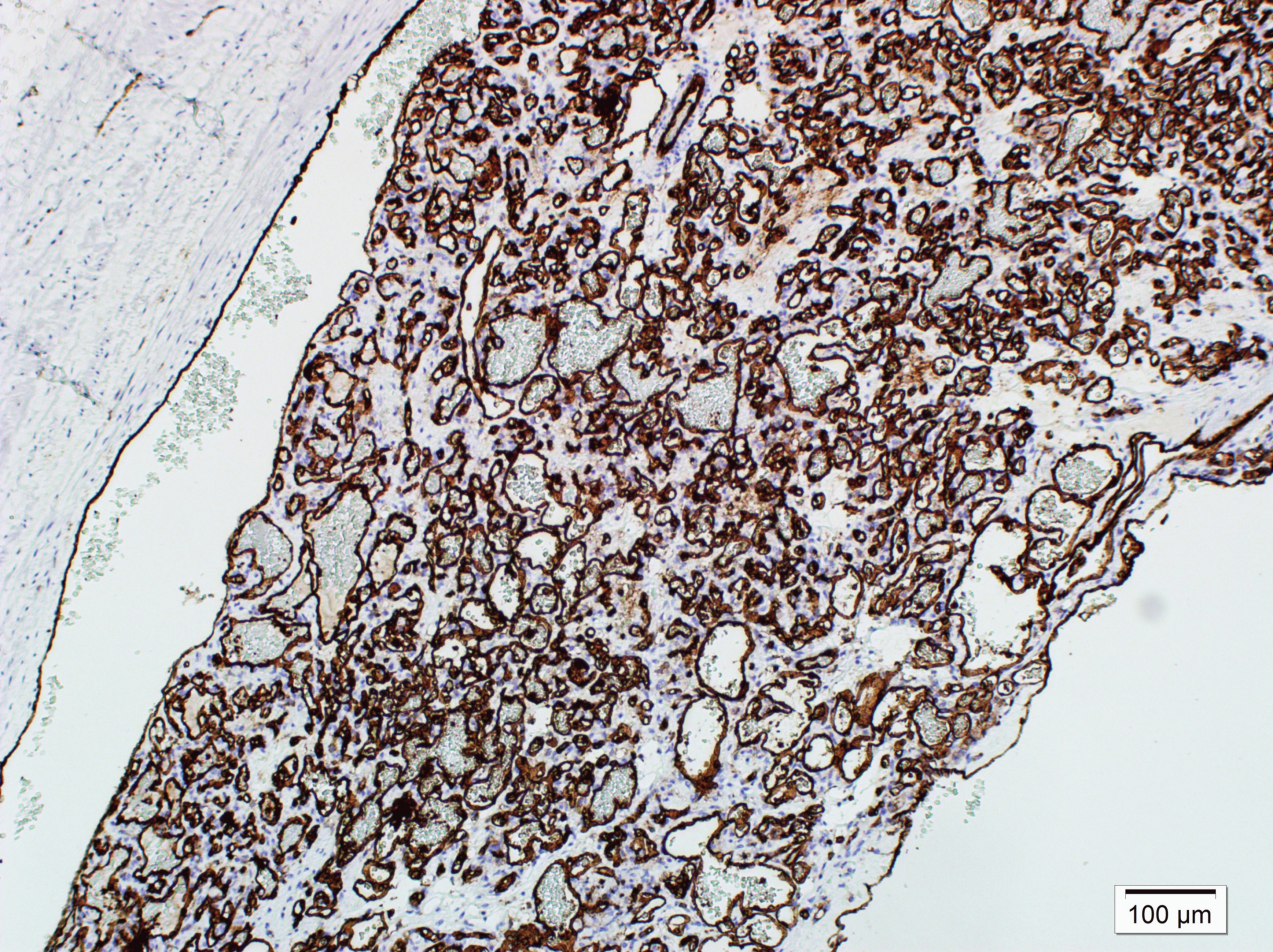
CD31 immunohistochemical staining at x100 magnification showing diffuse staining in endothelial cells.

Histological evaluation of the testis showed normal age-related morphology. In contrast, the epididymal mass was composed of dilated vascular structures lined by bland endothelial cells within a fibromyxoid stroma, characteristic of ILCH (Figure 3). The endothelial cells showed strong positivity for CD31, CD34, and ERG, confirming the vascular nature of the lesion (Figure 4). Smooth muscle actin (SMA) staining highlighted the pericytes surrounding the capillaries. No cellular atypia or mitotic figures were identified, ruling out malignancy. The lesion was diagnosed as an ILCH.

The patient was followed for 18 months after surgery, and no recurrence was observed.

## Discussion

ILCH is a rare vascular lesion with a benign course, but its unusual presentation can pose diagnostic challenges. Most reported cases occur in the head, neck, or upper extremities [2,4,8]. ILCH's rarity, particularly in the genital region, contributes to its frequent misdiagnosis as a malignant vascular tumor. To the best of our knowledge, only a few cases of ILCH involving the genital region have been reported, and this is the first case identified in the epididymis mimicking a testicular tumor. The patient’s preoperative imaging studies suggested malignancy due to the hypervascular nature of the lesion, a feature commonly associated with malignant tumors [1,3,4].

Histopathological examination and immunohistochemical staining were crucial for confirming the diagnosis of ILCH. The differential diagnosis of ILCH includes venous thrombosis, angiosarcoma, and intravascular papillary endothelial hyperplasia (Masson tumor). These entities share certain clinical and radiological features but can be distinguished by their histological architecture and immunohistochemical profiles. The lesion’s strong expression of CD31, CD34, and ERG, along with the presence of pericytes highlighted by SMA staining, supported the diagnosis of a benign vascular lesion [5,9]. The absence of atypia and mitotic activity further ruled out malignancy.

Surgical excision remains the treatment of choice for ILCH, as it prevents potential complications such as thrombosis or embolism. The benign nature of the lesion, along with the absence of cellular atypia and mitotic figures, supports the good prognosis associated with complete excision.

## Conclusions

This case highlights the importance of considering ILCH in the differential diagnosis of vascular tumors in uncommon locations. ILCH can easily be mistaken for a malignancy, as demonstrated by our case, where the lesion was misdiagnosed preoperatively as a testicular tumor. Careful histopathological examination is essential for the correct diagnosis and avoidance of unnecessary aggressive treatments.
